# The Disinfection of Wounds

**Published:** 1893-06-17

**Authors:** C. R. Illingworth

**Affiliations:** West Kensington, W.


### THE DISINFECTION OF WOUNDS.

SIR,—Permit me to point to the important experiments of Dr. Schimmelbush, detailed at the Surgical Congress, as indicating the necessity for further work upon the same lines. I am persuaded that in this way not only will the fallaciousness of the doctrine of phagocytosis be demonstrated, but also the validity of antiseptic surgery be clearly proved. The experiments showed that strong germicidal agents had no effect in arresting sepsis in infected wounds. I submit that the reason why they failed, lay in the fact that they were not employed in such a form as to place them on what may be styled an "equal footing" with the inoculating material. The invariable action of bacteria is one of a defibrinating or liquefying nature; whilst that of all the bactericides of any power in Dr. Schimmelbush's experiments is productive of fibrinous effusion in the blood and tissues. The consequence is, as was found, failure of disinfection and death of the subject.

What is needed in further experiments is a potent antiseptic applied to a living infected wound in such a form as will not excite the formation of albumen compounds and fibrinous effusions, to the detriment of its pursuing and bactericidal powers. The only strong antiseptic at present known in such a form and with such powers is the iodide of potassium or sodium solution of the biniodide of mercury. During the past seven years I have treated all kinds of poisoned wounds with it, and I have never known it fail in preventing and curing erysipelas and blood poisoning of every description.—I am Sir your obedient servant,

C. R. ILLINGWORTH, M.D.

West Kensington, W., June 6th, 1893.

